# Blad-Containing Oligomer Fungicidal Activity on Human Pathogenic Yeasts. From the Outside to the Inside of the Target Cell

**DOI:** 10.3389/fmicb.2016.01803

**Published:** 2016-11-14

**Authors:** Ana M. Pinheiro, Alexandra Carreira, Filipe Rollo, Rui Fernandes, Ricardo B. Ferreira, Sara A. Monteiro

**Affiliations:** ^1^Linking Landscape, Environment, Agriculture and Food, Instituto Superior de Agronomia, Universidade de LisboaLisboa, Portugal; ^2^CEV, SACantanhede, Portugal; ^3^Histology and Electron Microscopy Service, Instituto de Investigação e Inovação em SaúdePorto, Portugal

**Keywords:** *Candida albicans*, antifungal, natural product, pathogenic yeast, protein-based

## Abstract

Blad polypeptide comprises residues 109–281 of *Lupinus albus* β-conglutin precursor. It occurs naturally as a major subunit of an edible, 210 kDa oligomer which accumulates to high levels, exclusively in the cotyledons of *Lupinus* seedlings between the 4th and 14th day after the onset of germination. Blad-containing oligomer (BCO) exhibits a potent and broad spectrum fungicide activity toward plant pathogens and is now on sale in the US under the tradename Fracture^TM^. In this work we demonstrate its antifungal activity toward human pathogens and provide some insights on its mode of action. BCO bioactivity was evaluated in eight yeast species and compared to that of amphotericin B (AMB). BCO behaved similarly to AMB in what concerns both cellular inhibition and cellular death. As a lectin, BCO binds strongly to chitin. In addition, BCO is known to possess ‘exochitinase’ and ‘endochitosanase’ activities. However, no clear disruption was visualized at the cell wall after exposure to a lethal BCO concentration, except in cell buds. Immunofluorescent and immunogold labeling clearly indicate that BCO enters the cell, and membrane destabilization was also demonstrated. The absence of haemolytic activity, its biological origin, and its extraordinary antifungal activity are the major outcomes of this work, and provide a solid background for a future application as a new antifungal therapeutic drug. Furthermore, its predictable multisite mode of action suggests a low risk of inducing resistance mechanisms, which are now a major problem with other currently available antifungal drugs.

## Introduction

Fungal infections have become an important factor of morbidity and mortality and represent an increasing burden on medical systems (Del Poeta, 2010; Huffnagle and Noverr, 2013), being associated with unavoidable high mortality rates, similar to those caused by tuberculosis or malaria (Brown et al., 2012).

*Candida* species are regarded as common components of the body microbiota in healthy humans (Kathiravan et al., 2012) but are also responsible for candidaemia, an invasive fungal infection associated with substantial morbidity, mortality and healthcare costs (Zaoutis et al., 2005; Bassetti et al., 2015), being among the top ten pathogens causing bloodstream infections. Although *C. albicans* still remains the most abundant and significant species associated with the disease, other medically important species of *Candida* are rising (Klepser, 2011; Papon et al., 2013; Sardi et al., 2013; Won et al., 2015) including *C. glabrata, C. rugosa, C. parapsilosis, C. tropicalis, C. dubliniensis, C. krusei*, and *C. lusitaniae* (Kathiravan et al., 2012; Huffnagle and Noverr, 2013; León et al., 2014). A number of factors may explain this gradual change in epidemiology, such as severe immunosuppression or illness, prematurity, exposure to broad-spectrum antibiotics and older patients (Sardi et al., 2013).

Some microorganisms are naturally resistant to certain types of antifungal medications while other species, although susceptible to a particular type of medication, have been registered as developing resistance over time as a result of improper antifungal use (Lortholary et al., 2011; Shah et al., 2012). Therefore, new formulations of antifungals, combination therapies and development of new bioactive compounds may be the key for a better therapeutic outcome (Spampinato and Leonardi, 2013), especially considering that antifungal research is stagnant, when compared to other pathologies. In the last decades, just a few new antifungal agents were unveiled and they were mainly based on the structural modification of already discovered drugs (Rubbiani et al., 2016).

Conventional therapies against fungi rely on a very limited number of drugs (Myung and Klittich, 2015; Rubbiani et al., 2016), from four different classes, and their antifungal activity and mode of action is well reviewed in the literature: polyenes (Patterson, 2006; Palacios et al., 2007; Denning and Hope, 2010; Cornely et al., 2012; Mesa-Arango et al., 2014; Nett and Andes, 2016), azoles (Odds et al., 2003; Chapman et al., 2008; Cui et al., 2015), echinocandins (Spampinato and Leonardi, 2013; Cui et al., 2015), and pyrimidine analogs (Spampinato and Leonardi, 2013; Nett and Andes, 2016; Prasad et al., 2016). Despite the introduction of new antifungal agents, the clinical outcomes for most invasive fungal infections are far from ideal (Roemer and Krysan, 2014). It is therefore imperative to continue the search for different strategies to combat fungal infections (Spampinato and Leonardi, 2013; Garrigues et al., 2016; Prasad et al., 2016).

Antimicrobial peptides and proteins are produced by multicellular organisms as a defense mechanism against competing pathogenic microbes (Selitrennikoff, 2001) and have been considered as candidates for the development of novel antimicrobial compounds (Fjell et al., 2012; Virágh et al., 2015; Garrigues et al., 2016). A main hurdle that has hindered the development of both natural and non-natural antimicrobial peptides and proteins as therapeutic agents is the fact that many of them exhibit antifungal activity *in vitro* (e.g., magainin), but are only effective *in vivo* at very high, often toxic, levels (Darveau et al., 1991; Zasloff, 2002). An additional difficulty associated to the potential use of proteins is their inherent typical instability. Currently there are only five antifungal peptides recorded as having reached the clinical stage of the drug development cycle (Duncan and O’Neil, 2013). The most prominent group within the antifungal peptides are the defensins from plants, insects and mammals (Hegedüs and Marx, 2013). Plant defensins, like Psd1 (Lobo et al., 2007), Nad1 (Van Der Weerden et al., 2008) and MtDef4 (Sagaram et al., 2013) specifically interact with fungal membrane sphingolipids and phospholipids, enter the cell and interfere with nuclear and cytosolic proteins (Vriens et al., 2016).

A remarkable, novel antifungal 20.4 kDa polypeptide was recently described. It is the major subunit of a 210 kDa glyco-oligomer, termed Blad-containing oligomer (BCO), which accumulates abundantly in *Lupinus albus* cotyledons between days 4 and 12 after the onset of germination. The BCO appears to reunite in a single molecule several selected characteristics, making it a versatile, multifunctional protein (Monteiro et al., 2010). Its extreme resistance to chemical inactivation but high susceptibility to proteolytic attack (Monteiro et al., 2015), associated to a powerful and broad spectrum antifungal activity toward plant pathogens makes it a unique, flexible and environmental friend active ingredient, now on sale in the US under the tradename Fracture^TM^.

In this work we demonstrate that the BCO also has a higher inhibition potency for human pathogens than the azoles and similar to amphotericin B (AMB) on a molar basis (Monteiro et al., 2015), making the BCO a very promising clinical antifungal agent. We also provide some insights on its highly complex and multitarget mechanism of action.

## Materials and Methods

### Microorganisms

Eight yeast strains were used, six belonging to *Candida* spp., one *Cryptococcus neoformans* strain (CBS 132) and one *Saccharomyces cerevisiae* strain (W303). *Candida* strains used were *C. albicans* var. *albicans* (CBS 562), *C. dubliniensis, C. glabrata, C. lusitaneae, C. parapsilosis* (PYCC 2597) and *C. tropicalis*. [CBS – Centraalbureau voor Schimmelcultures; PYCC – Portuguese Yeast Culture Collection; The other strains were a kind gift of Institute of Microbiology, Faculty of Medicine of the University of Coimbra (Paulo et al., 2009)]. All yeasts were grown at 35°C for 24 h, except for *C. neoformans* that was grown for 72 h, in Glucose Yeast Peptone (GYP) medium [0.5% (w/v) peptone, 0.5% (w/v) yeast extract, 2% (w/v) glucose, 1.5% (w/v) agar]. For the different experiments performed, three media were used; RPMI 1640 (Applichem), pH 7.0, supplemented with 2.08% (w/v) glucose and 6.9% (w/v) MOPS [3-(*N*-morpholino)propanesulfonic acid]; YNB (Difco), pH 7.0, supplemented with 2% (w/v) glucose and 0.1% (w/v) MOPS; and PDB (DIFCO), buffered at pH 7.5.

### *Lupinus albus* and BCO Purification

Dry seeds of *Lupinus albus* were germinated and grown in growth chambers with a photoperiod of 16 h light/8 h dark at 18°C, for periods up to 10 days. The seed coats were removed and the intact cotyledons were dissected from the axes and stored frozen at -80°C until needed. BCO is a breakdown product of β-conglutin catabolism, and it was extracted and isolated from the cotyledons of 8-days old seedlings as described by (Monteiro et al., 2015). The protein corresponding to β-conglutin was purified by AKTA anion exchange chromatography followed by AKTA gel filtration chromatography as follows: the total globulin fraction was loaded on the Q-Sepharose column (Ø = 1 cm; h = 8 cm; flow rate = 1.5 mL/min) previously equilibrated in 20 mM Tris-HCl buffer, pH 7.5, and eluted with a linear gradient of NaCl (0 to 1 M). The fraction containing the BCO, eluted between 0.25 and 0.35 M NaCl, was subsequently subjected to gel filtration on an AKTA Superose 12 HR 10/30 column (GE Healthcare Life Sciences), equilibrated in 0.1 M Tris-HCl buffer (pH 7.5). This last purification step does not affect the polypeptide pattern of the protein, but removes unidentified low molecular mass compounds, resulting in a high pure BCO sample.

### Antifungal Agents

The BCO was extracted and purified as described above and stored lyophilized at room temperature (RT). AMB was obtained from their respective manufacturers and stock solutions were prepared and stored frozen at -20°C until used.

### Production of BCO Polyclonal Antibodies

A sample of BCO was lyophilized and ressuspended in Freund adjuvant. New Zeland female rabbits and rats were immunized with the purified BCO sample. To obtain a high titer, three boosters injections of 100 μg/mL of antigen each were given every 2 weeks in complete Freund’s diluted 1:10 with incomplete adjuvant. Total blood was taken from the heart 12 days after the third booster injection. Blood samples were allowed to clot, and the serum was collected, centrifuged, and stored at -70°C. To purify the IgG present in the serum, a chromatography in a Protein G sepharose column was conducted.

### Antifungal Susceptibility Tests

Susceptibility tests were made according to the CLSI – Clinical and Laboratory Standards Institute (former NCCLS – National Committee for Clinical Laboratory Standards) guideline M27-A2 (NCCLS, 2002) with some adjustments, using the broth microdilution method. Yeast cells were grown on GYP medium and the inoculum suspension was prepared by picking fresh colonies and resuspending them in 5 mL of sterile 0.9% (w/v) saline (NaCl). The resulting suspension was vortexed for 15 s and the cell density was adjusted with a spectrophotometer to give an inoculum concentration of 10^6^ cells per mL. The final inoculum suspension was prepared by a 1:50 dilution followed by a 1:20 dilution with double-strength broth medium, which resulted in a final concentration of 10^3^ cells per mL. One other final inoculum concentration was tested in *C. albicans*, 10^5^ cells/mL, achieved by a 1:10 dilution with double-strength broth medium, for allowing a sufficient number of cells to be visualized under the microscope and to enable the determination of a MFC based on a 99.9% killing (see Minimum Fungicidal Concentration section below). The inoculum size was verified by enumeration of CFUs obtained by subculturing on GYP plates. The solution of the BCO was prepared in ultrapure sterile water and 200 μL were added to the first line of the microplate. A serial two-fold dilution was made, twelve times, using ultrapure sterile water, in the 96-well microplates. The final concentration of the BCO, after addition of the inocula, ranged from 0.002 to 4.762 μM when using PDB medium and from 0.012 to 23.81 μM with RPMI medium. The serial twofold dilutions of AMB ranged from 0.03 to 17.31 μM. The yeast inoculum (100 μL) was added to each well of the microplate, containing 100 μL of the drug solution (twofold concentrated). The final volume in each well was 200 μL. The microplate was incubated at 35°C and examined after 72 h. Minimum inhibitory concentrations (MICs) are the lowest drug concentration showing absence of growth, as recorded visually. To evaluate the effect of sorbitol on the fungal susceptibility to the BCO, the growth medium (twofold concentrated) was supplemented with 2.4 M sorbitol (final concentration 1.2 M). All these tests were performed with three different batches of the BCO (triplicates).

### Minimum Fungicidal Concentrations (MFCs)

Minimum fungicidal concentrations were determined according to (Espinel-Ingroff, 1998). After MIC determination, as previously described, 30 μL aliquots were subcultured from each well that showed no visual growth onto GYP plates. This procedure was performed to minimize drug carryover effects (Espinel-Ingroff, 1998). The plates were incubated at 35°C for 24 h. All these tests were performed with three different batches of the BCO (triplicates).

The non-existence of a standard method for determining MFCs in yeasts as led to an indiscriminate use of a wide range of methodologies for determining this value (Vazquez et al., 1997; Johnson et al., 1998; Espinel-Ingroff, 2001). To maintain the standardized methodology for determining MIC, an initial inoculum of 10^3^ CFU/mL was used, although it does not allow the detection of 99.9% killing. To minimize this restriction, the MFC considered in this study was the lowest drug concentration where no growth was observed after plating 30 μL on GYP plates (0 CFUs). For achieving a 99.9% killing an initial inoculum size of 10^5^ CFU/mL was tested for *C. albicans* only.

### Time-Kill Curves

The effect of the BCO on the growth of human fungal pathogens was evaluated by using *C. albicans* as a model organism and by comparing the results observed with those of AMB, in PDB pH 7.5. The assays were conducted in the presence of different BCO and AMB concentrations. A cell suspension was grown overnight in 20 mL of PDB pH 7.5, at 35°C, 150 rpm and refreshed in 20 mL of PDB pH 7.5, approximately 5 h before addition to the culture medium. To obtain an initial concentration of approximately 10^5^ CFU/mL, the OD_640 nm_ was adjusted to 0.1 and then 10-fold diluted with PDB pH 7.5, to a final volume of 100 mL, in 500 mL Erlenmeyer flaks. The cultures were incubated at 35°C without shaking. At regular intervals, samples were collected for absorbance measurements, viable cell counts and morphological evaluation. For viable cell counts, 30 μL aliquots of the culture were taken, diluted if needed, and plated on GYP agar plates. Each time-kill curve was performed in triplicate and a representative curve is shown in the results.

### Effect of the BCO on Yeast Cell Volume

The effect of the BCO on the yeast cell volume was evaluated using *S. cerevisiae* (W303) as a yeast model. Two yeast cultures were grown as described in the Time-kill curves section, one in the presence of the BCO and the other kept as control. At regular intervals, samples were collected and an estimate of the cell volume was made, by measuring the diameter of 100 cells. The cell volume was calculated considering the shape of the cells as a sphere. At each sampling time the average volume of the 100 cells was calculated as well as the corresponding standard deviation.

### Haemolytic Activity

The haemolytic activity of the BCO was analyzed according to (Ling et al., 2015). Briefly, fresh red blood cells from rabbit were collected and washed with PBS until the upper phase was clear after centrifugation. The pellet was ressuspended in PBS to an OD_600 nm_ = 24 and added to a 96-well microplate. The solution of the BCO was prepared in ultrapure sterile water and a serial twofold dilution was made in water and added to the wells. The final concentration of the BCO ranged from 0.04 to 4.76 μM. After 1 h incubation at 37°C, cells were centrifuged at 1000 *g* and the supernatant was diluted and measured at 450 nm in a BioTek’s Take3^TM^ Multi-Volume Plate spectrophotometer.

### Viability Assessments

The LIVE/DEAD^®^ Yeast Viability Kit (Molecular Probes) was used to evaluate fungal viability. A FUN1 100 μM working solution was prepared in 10 mM MOPS buffer, pH 7.2, with 2% (w/v) glucose. A 50 μM calcofluor white working solution was prepared in distilled water. Forty microliter of fungal culture and 5 μL of FUN1 working solution were mixed thoroughly and incubated at 30°C in the dark. After 30 min, 5 μL of calcofluor white working solution were added to the culture and mixed thoroughly. Five microliter of the cell culture were trapped between a microscope slide and a coverslip for visualization on a fluorescence microscope.

### Cell Membrane Integrity

After 24 h incubation with the BCO, under the same conditions as described in the Time-kill curves section, cells were incubated with propidium iodide at a final concentration of 7.5 μM, for 10 min at 4°C. Five microliter of the cell culture were trapped between a microscope slide and a coverslip, for visualization on a fluorescence microscope.

### Morphological Changes in *C. albicans* Cells in the Presence of the BCO

Morphological changes in *C. albicans* cells in the presence of the BCO were assessed by Transmission Electron Microscopy (TEM). The culture was prepared and kept under the same set of conditions as previously described in the Time-kill curves section. At regular intervals, samples were collected, washed twice with saline [9.5% (w/v) NaCl] and concentrated by centrifugation (3500 *g* for 10 min) to a final concentration of 1 to 5 × 10^6^ CFU/mL. Cell concentration was confirmed at all points by plating in GYP agar. The samples were collected and fixed in 2% (v/v) glutaraldehyde and 2.5% (v/v) paraformaldehyde in 0.1 M sodium cacodylate buffer pH 7.4. Then, they were post fixed in 2% (w/v) OsO_4_, dehydrated and embedded in epon. The ultrathin-sections (60 nm) were counterstained with aqueous uranyl acetate solution and lead citrate. Controls were prepared under the same conditions, but in the absence of the BCO.

### Immunolocalization of the BCO

Immunolocalization of the BCO was accomplished by both indirect immunofluorescence and immunogold methods. In both cases the culture was prepared and kept under the same conditions as previously described in the Time-kill curves section.

Immunofluorescency studies were accomplished according to (Lawrence et al., 2004) with some modifications. After 24 h incubation with a lethal concentration of the BCO, the culture was concentrated by centrifugation (3500 *g* for 10 min) to a final concentration of 1 to 5 × 10^7^ CFU/mL followed by treatment with lyticase (0.4 mg/mL in 500 mM Tris-HCl, 1 M sorbitol, 0.8 M KCl, 10 mM MgS0_4_, pH 7.5) for 2 h at 30°C, to digest the cell wall, allowing the subsequent cell membrane permeabilization. The culture was washed with PBS (137 mM NaCl, 1.5 mM KH_2_PO_4_, 8.1 mM Na_2_HPO_4_ and 2.7 mM KCl) followed by fixation in 4% (v/v) formaldehyde, for 30 min, at 30°C. After two washes with PBS and PBS containing 0.1% (v/v) Triton X-100 for cell membrane permeabilization, cells were blocked with bovine serum albumine (BSA) 5% (w/v) in PBS containing 0.1% (v/v) Triton X-100 for 30 min. Cells were washed with PBS and incubated with the first antibody (anti-BCO), produced in rabbit and diluted 1:500 in PBS containing 0.1% (v/v) Triton X-100 and 0.1% (w/v) BSA, for 16 h at 4°C. The cells were then washed in PBS and incubated with a second, anti-rabbit antibody, produced in goat, conjugated with FITC and diluted 1:80 in PBS with 1% (w/v) BSA, for 1 h at 37°C. After washing twice with PBS for 15 min, 50 μM calcofluor white were added and 5 μL of the cell culture were trapped between a microscope slide and a coverslip, for visualization on a confocal microscope.

For immunogold analysis, the samples were collected after 6, 12, and 24 h of incubation with the BCO and subsequently fixed in 0.1% (v/v) glutaraldehyde in 0.1 M sodium cacodylate buffer pH 7.4 for 1 h. Then, they were dehydrated and embedded in LRWhite. Thin sections on TBS were immunolabeled after incubation on etching process: grids were incubated in a humid chamber in large drops of a saturated aqueous solution of sodium metaperiodate, for 1 h at RT. After washing the grids, they were first incubated for 20 min in 2% (w/v) gelatin in TBS, and a second 5 min incubation was done with 15 mM glycine. The grids were blocked with a solution containing 2% (w/v) immunoglobulin-free BSA. Sections were then incubated overnight (16–18 h) with the first antibody (anti-BCO) diluted 1:250 in TBS containing 2% (w/v) BSA and 1% (v/v) Tween-20/3% (w/v) NaCl. The grids were then washed by floating them on drops of 0.1% (w/v) BSA /TBS (four changes, 2 min each) followed by 20 min incubation on TBS with 1% (w/v) BSA. Bound antibodies were visualized by incubating the sections for 1 h with the second antibody-gold conjugate (10 nm diameter particles) diluted 1:25 in PBS with 1% (w/v) BSA, 1% (v/v) TBS. Finally, grids were washed on drops of water (six changes, 10 min each). The immune-complexes formed were visible as little black dots when observed by TEM.

### Microscopy

(I) *Fluorescence microscopy*. Samples were observed under a fluorescence microscope (Axioscope A1 with phase contrast and epi-fluorescence, Zeiss) equipped with a camera (AxioCam ICm1, Zeiss), using three different filters: Filter Set 49 DAPI (Excitation G 365, Emission BP 420/470); Filter Set 10 FITC/GFP (Excitation BP 450-490, Emission BP 515-565) and Filter Set 15 Rodhamine (Excitation BP 540-552, Emission LP 590). (II) *Confocal microscopy*. The images were acquired with a Leica TCS SP5 II confocal microscope equipped with an objective HCX PL APO CS 63x/1.3 Glycerol (Leica Microsystems, Germany). Samples were excited by 488 nm laser line and emitted signal detected in the range of 496-564 nm with a HyDet detector (fluorescence channel) and a PMT detector (transmission channel). Images were acquired with 512 × 512 pixels and a pixel size of 68 nm. (III) *Transmission electron microscopy*. The sections were examined under a JEOL JEM 1400 TEM 120kV (Tokyo, Japan). Images were digitally recorded using a CCD digital camera Orious 1100W Tokyo, Japan.

## Results

### Determination of Minimum Inhibitory and Fungicidal Concentrations

The antifungal activity of three different batches of the BCO was evaluated in six strains belonging to six *Candida* species and in one strain of *C. neoformans*, with each strain cultured in two different growth media. *C. albicans* CBS 562 was also tested for resistance to AMB, one commonly used antifungal drug. The MICs and MFCs of the BCO are shown in **Table 1**. MIC values in RPMI medium for the entire group varied from 0.19 to 2.98 μM. Regarding the MFCs of the BCO, the values obtained were higher than the highest concentration tested. The exception was the strain of *C. albicans*, for which the MFC of the BCO was 23.81 μM (**Table 1**).

**Table 1:** Ranges of MIC and MFC values of the BCO for various yeast species grown in two different culture media, with an initial inoculum of 10^3^ CFU/mL and tested with three different batches of the BCO.

	RPMI medium		PDB medium	
Yeast Species	MIC (μM)	MFC (μM)	MIC (μM)	MFC (μM)
*Candida albicans*	1.49	23.81	0.15–0.31	0.31–0.61
*Candida dubliniensis*	0.75–1.49	>23.81	0.15–0.31	0.31–0.61
*Candida glabrata*	0.19–0.37	>23.81	0.08–0.15	0.31–0.61
*Candida lusitaneae*	0.37	>23.81	0.15–0.31	0.61
*Candida parapsilosis*	0.37	>23.81	0.15–0.31	2.38
*Candida tropicalis*	2.98	>23.81	0.15	0.61
*Cryptococcus neoformans*	0.37	>23.81	0.08–0.15	0.15–0.31

When the antifungal activity of the BCO was assessed in PDB medium, the MIC and MFC values obtained were more consistent among the different species, which is in accordance with previous studies that demonstrated that PDB is a more suitable medium for testing the bioactivity of this oligomer (Monteiro et al., 2015). All strains tested presented a similar susceptibility to the BCO, with MIC values ranging from 0.08 to 0.31 μM (**Table 1**). Regarding the MFCs of the BCO in PDB medium, the values varied from 0.15 to 2.38 μM. For the strains of *C. albicans, C. dubliniensis* and *C. neoformans*, the MFCs were always only twice of the respective MICs and for those of *C. glabrata* and *C. lusitaneae* were always four times higher. The MFC of BCO for *C. parapsilosis* was too much higher than the other ones (2.38 μM) (**Table 1**). Overall the results obtained for the BCO are indicative of a potent antifungal activity for these fungal species.

The antifungal activity of the BCO was then compared to that exhibited by AMB, in PDB medium, using *C. albicans* as a control model. This specie has long been used as a model in several fungal research studies (Fu et al., 2008; Kabir et al., 2012), and also showed a strong susceptibility to the BCO, as demonstrated by the corresponding MIC and MFC values (**Table 1**). Since the optimum inoculum density for the subsequent microscopy tests was found to be 10^5^ CFU/mL, and since this concentration allows the detection of a 99.9% killing based MFC, both minimum inhibitory and fungicidal concentrations were determined with this inoculum size. The results are presented in **Table 2**. As expected, there was an increase in MIC and MFC values, due to a higher number of cells in the initial inoculum. However, these results are still consistent with those obtained before (**Table 1**). The concentration of the BCO needed to induce death of 99.9% of the microorganisms was only twice the MIC (0.60–1.19 and 1.19–2.38 μM, respectively). The same effect was observed for AMB (MIC and MFC values of 1.1 and 2.2 μM, respectively), which is also consistent with the values reported in the literature (Manavathu et al., 1998; Spreghini et al., 2012).

**Table 2:** Ranges of MIC and MFC values of the BCO and AMB for *C. albicans* (in PDB medium at pH 7.5), with an initial inoculum of 10^5^ CFU/mL and tested with three different batches of the BCO.

Antifungal agent	MIC (μM)	MFC (μM)
BCO	0.60–1.19	1.19–2.38
AMB	1.1	2.2

### BCO Leads to an Increase on the Yeast Cell Volume Unrelated to Major Cell Wall Disturbances

**FIGURE 1:**
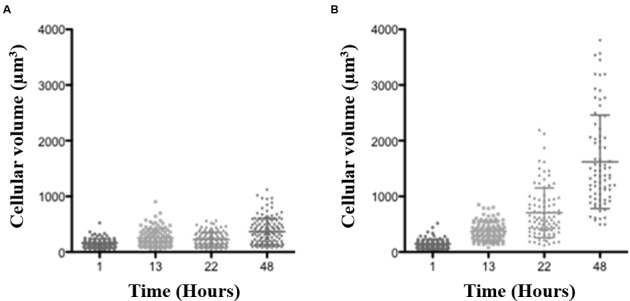
**Blad-containing oligomer (BCO) effect on *S. cerevisiae* W303 cell volume along time.** *S. cerevisiae* cells were grown in YNB medium supplemented with 2% (w/v) glucose, pH 7.0, at 30°C. The horizontal bars show the mean values (central bar) and the standard deviation (edge bars). **(A)** – culture kept without the BCO; **(B)** – culture with 0.152 μM BCO.

Given the demonstrated affinity of the BCO to chitin (Monteiro et al., 2015), two studies were conducted for assessing its possible effect in cell wall biosynthesis. *S. cerevisiae* was used as the model organism for these studies. The cells were exposed to BCO at the MIC value and an estimate of the cell volume at three-time sampling points was made. The results obtained are described in **Figure 1** and show that the presence of the BCO leads to a progressive increase in the average volume of the cells. The volume variation between the first and the last sample analyzed is higher than 10 fold (147 μm^3^ after 1 h of incubation and 1621 μm^3^ after 48 h).

Considering the results above, if the BCO mode of action is at least in part based on cell wall damage, it is expected for the cell to suffer an increase of volume under hypotonic conditions, until eventually bursts. If this is the case, under isotonic conditions, or even in a slightly hypertonic medium, this effect should not occur, since there is no water entering into the cells. With the purpose of testing if the osmotic stabilizer sorbitol counteracts the toxicity of the BCO, 1.2 M sorbitol was added to the culture medium (creating a slightly hypertonic condition). Tests were carried out in the absence and in the presence of 1.2 M sorbitol in *S. cerevisiae.* The results demonstrated that the presence of 1.2 M sorbitol in the culture medium did not reduce the antifungal effect of the BCO, since the MIC values were the same in both cases (0.15 μM). This result suggests that ultimately, the toxicity of the BCO to fungi is not dependent on dramatic changes in cell wall integrity despite its ability to bind very tightly to the chitin polymer (Monteiro et al., 2015).

### BCO Has a Dose-Dependent Effect on the Growth of *C. albicans*

**FIGURE 2:**
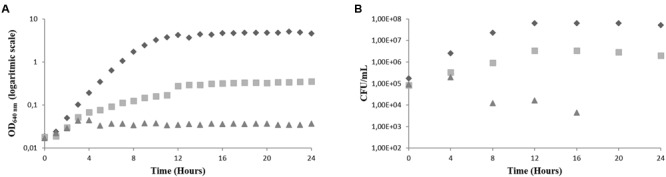
**Effect of the BCO on the growth of *C. albicans* in PDB medium, pH 7.5, 35°C, without agitation (representative curve of triplicate experiments). (A)** OD_640 nm_ **(B)** CFU/mL. BCO concentration in the culture medium: 0 μM (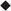), 1.19 μM (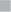) and 2.38 μM (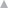).

**FIGURE 3:**
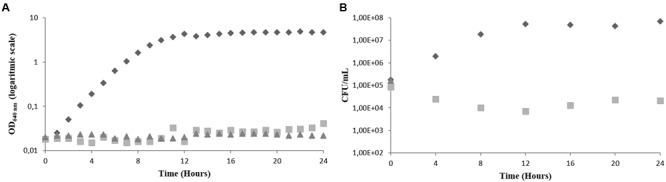
**Effect of AMB on the growth of *C. albicans* in PDB medium, pH 7.5, 35**°**C, without agitation (representative curve of triplicate experiments). (A)** OD_640 nm_ **(B)** CFU/mL. AMB concentration in the culture medium: 0 μM (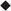), 1.1 μM (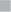) and 2.2 μM (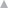).

Determination of the “killing” of an isolate over time by one or more antimicrobial agents under controlled conditions is known as the time-kill method (Pfaller et al., 2004). It is a broth based method where the rate of killing of a fixed inoculum is determined by sampling control (organism, no drug) and antimicrobial agent-containing tubes or flasks, at certain time intervals, and determining the survivor colony count (CFU/mL) by spreading each sample onto an agar plate. In order to study the effect of the BCO on the growth of *C. albicans*, time-kill curves were performed in PDB medium. Several samples were taken during these experiments in order to assess the evolution of the number of viable cells (OD_640 nm_ and CFU counts). Two concentrations of BCO were used, 1.19 and 2.38 μM, corresponding to the minimum inhibitory and fungicidal concentrations, respectively, as determined previously (**Table 2**). A fraction of the culture grown under the same conditions but without BCO was tested for control purposes. The results are shown in **Figure 2** for the BCO and in **Figure 3** for AMB.

**Figure 2** shows that the addition of BCO to the culture medium had a strong effect in the growth of *C. albicans.* It is possible to observe that the culture grown in the absence of BCO presents a normal growth curve, being in the exponential phase of growth for approximately 12 h, before entering the stationary phase. This was observed by following both OD_640 nm_ readings and CFU/mL counts. Cells grown in the presence of the MIC of the BCO, showed a decrease in the growth rate when compared to the control, which resulted in a lower final optical density (**Figure 2A**) and a lower final CFU/mL count (**Figure 2B**). This result indicates that the concentration of the BCO tested had, indeed, the ability to inhibit the growth of this microorganism. Cells grown in the presence of the minimum fungicidal concentration became non-viable after 16 h of growth. This was observed by both stabilization of OD_640 nm_, just after 4 h of incubation (**Figure 2A**), after a slight initial growth, and absence of CFU counts (**Figure 2B**).

The same assay was performed using AMB as the antifungal agent. Two different concentrations were also used, 1.1 and 2.2 μM, corresponding to the minimum inhibitory and fungicidal concentration, respectively. The results obtained were very similar to those obtained for the BCO and are shown in **Figure 3**. The control fraction stayed in exponential phase for 12 h, and the fraction exposed to the MIC showed a total absence of growth. When using the minimum fungicidal concentration of AMB cells became non-viable in the first 4 h of exposure, which was observed by the absence of CFU counts (**Figure 3B**). The prompt reduction in *C. albicans* viability caused by AMB is in accordance with the data published in the literature (Cantón et al., 2004; Leite et al., 2014). The major difference between the BCO and AMB in terms of killing kinetics is that both fungistatic and fungicidal activities of AMB, under these conditions, act more rapidly than those of the oligomer.

### The MFC of the BCO Induces a Severe Decrease on the Metabolic Activity in *C. albicans*

**FIGURE 4:**
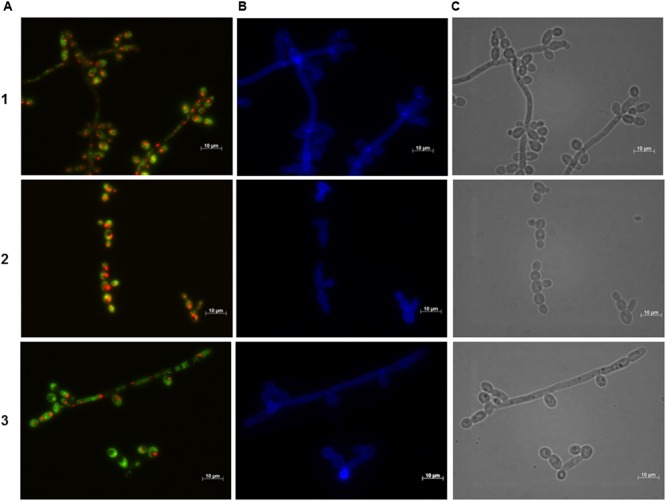
**Effect of the BCO on the metabolic activity and cellular integrity of *C. albicans* cultivated in PDB medium, pH 7.5, at 35°C, without agitation.** Samples were taken after 4 h of incubation. Concentration of the BCO in the culture medium: 1 – 0 μM, 2 – 1.19 μM, 3 – 2.38 μM. Labeling with FUN-1 **(A)**, calcofluor white **(B)**, and bright field microscopy **(C)**. Bar corresponds to 10 μm.

**FIGURE 5:**
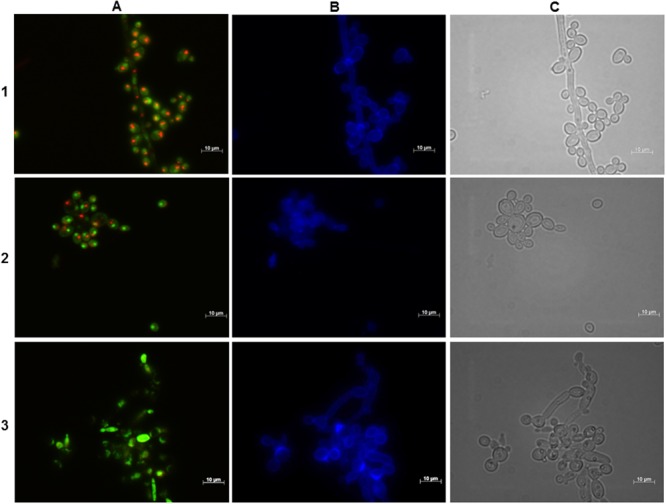
**Effect of the BCO on the metabolic activity and cellular integrity of *C. albicans* cultivated in PDB medium, pH 7.5, at 35°C, without agitation.** Samples were taken after 16 h of incubation. Concentration of the BCO in the culture medium: 1 – 0 μM, 2 – 1.19 μM, 3 – 2.38 μM. Labeling with FUN-1 **(A)**, calcofluor white **(B)**, and bright field microscopy **(C)**. Bar corresponds to 10 μm.

The effect of the BCO on the viability and cellular integrity of yeasts was evaluated using *C. albicans* as model and was assessed using samples collected along the growth curve, in PDB medium, under three different conditions: without drug (control), with the inhibitory (MIC) concentration (1.19 μM) and with the lethal (MFC) concentration (2.38 μM). Each sample was stained with FUN-1 and calcofluor white and visualized in a fluorescence microscope. FUN-1 binds to nucleic acids producing a yellowish green fluorescence in death cells with a damaged membrane. Cells without metabolic activity but with an intact plasma membrane also present a diffuse green coloration in the cytoplasm. On the other hand, in metabolically active cells, formation of orange cylindrical structures designated CIVS (Cylindrical IntraVacuolar Structures) is observed inside vacuoles. CIVS formation only occurs in metabolically active cells with an intact plasma membrane, meaning they are not observed in dead cells (Chew et al., 2015). Calcofluor white is a compound with high affinity to chitin and is normally used as a marker of the fungal cell wall. The results are shown in **Figures 4** and **5**.

**Figure 4** suggests that during the first 4 h of incubation with the BCO there are no changes regarding the viability and integrity of the cells, for all conditions tested, since the presence of CIVS indicates metabolic activity and the fluorescence with calcofluor white is normal and equal to the control fraction, indicating cell wall integrity. These results are consistent with the ones obtained in the growth curves (**Figure 2**).

After 12 h of incubation with the BCO, the control fraction continued to exhibit CIVS in the majority of the cells, confirming that they were still metabolically active. The fraction incubated with an inhibitory concentration of the BCO showed a slight lower number of cells with CIVS, meaning that some cells were metabolically active and, therefore viable and culturable, thus explaining the small increase in the OD_640 nm_ and CFU counts observed in the curve of **Figure 2**. At 12 h, cells incubated with the lethal concentration of the BCO presented very few CIVS, corresponding to a decrease in the metabolic activity (data not shown). This explains the decrease also observed in the CFU counts observed in **Figure 2**.

Microscopical observations performed at 16 h of incubation revealed to be a turning point in cell viability, which is in accordance to what is also observed with other antifungal drugs (Kim et al., 2011). Although both the control and MIC fractions of the BCO showed no changes as compared to the previous time point studied, the culture incubated with a lethal concentration of the BCO no longer presented visible CIVS. Only a diffuse green coloration in the cytoplasm was visible, corresponding to the absence of metabolic activity (**Figure 5**). However, when cultivated in a free BCO medium some cells were still able to grow (**Figure 2B**). At 24 h of incubation, the last time point studied, the control fraction presented some cells without CIVS, typical of an old culture and the MIC fraction presented even fewer metabolically active cells than in the previous time point studied (data not shown). This is in accordance with the stabilization of OD_640 nm_ and with the smaller number of culturable cells observed in the growth curves (**Figure 2**). The results obtained with the lethal concentration of the BCO were similar to those obtained after 16 h (cells without any metabolic activity), but at this point there were also no records of culturable cells (**Figure 2B**). This means that approximately between 16 to 24 h of incubation with a lethal concentration of the BCO, *C. albicans* lost the ability to grow in a free BCO medium, and that the number of culturable cells was beneath the detection limit of the method (<10 CFU/mL). These results suggest that after 16 h of incubation with a lethal concentration of the BCO, cells are metabolically inactive (**Figure 5**), non-viable and non-culturable (**Figure 2**). During these periods, the integrity of the cell wall remained unchanged regardless of the concentration of the BCO tested, as showed by calcofluor white staining (**Figures 4** and **5**).

### BCO Induces Cell Membrane Damages

**FIGURE 6:**
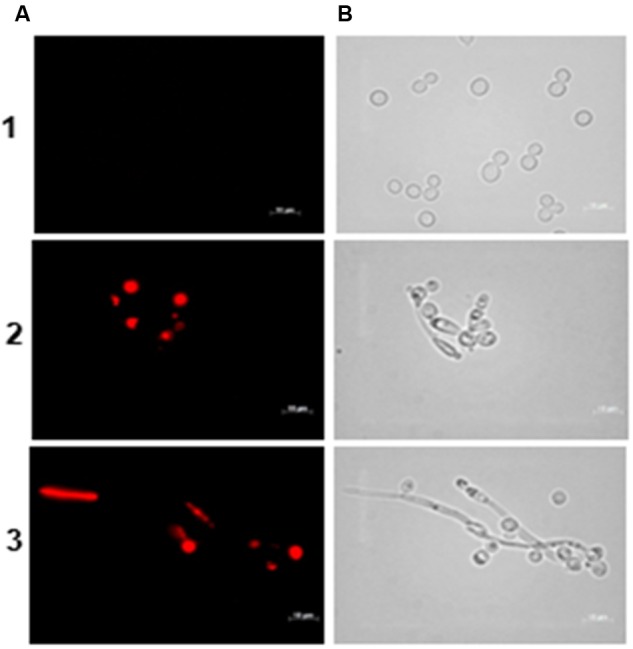
**Effect of the BCO on the viability and membrane integrity of *C. albicans* cultivated in PDB medium, pH 7.5, at 35°C, without agitation.** Samples were taken after 24 h of incubation. Concentration of the BCO in the culture medium: 1 – 0 μM, 2 – 1.19 μM and 3 – 2.38 μM. Labeling with propidium iodide **(A)**, and bright field microscopy **(B)**. Bar corresponds to 10 μm.

Cell membrane integrity was evaluated with propidium iodide. This compound binds to DNA and RNA producing a red fluorescence when intercalated with nucleic acids. However, due to its positive charge, it cannot cross an intact cell membrane and, therefore, only dead cells or cells with a damaged membrane are stained. Propidium iodide staining was evaluated at 24 h of incubation of *C. albicans* with the inhibitory (MIC) and with the lethal (MFC) concentration of the BCO, as determined previously (1.19 and 2.38 μM, respectively). The results, shown in **Figure 6**, clearly indicate that the BCO somehow destabilizes the plasma membrane, enabling the entrance of the fluorescent dye into the cell.

### BCO Moves from the Cell Wall into the Interior of the Cell

**FIGURE 7:**
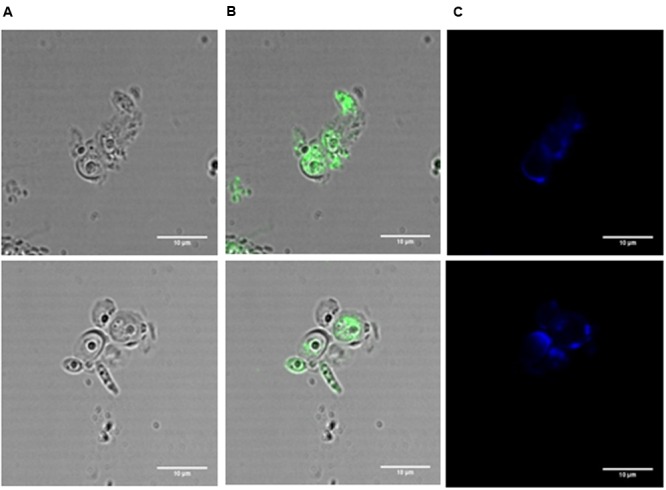
**Immunofluorescence in *C. albicans* incubated with a lethal concentration of the BCO for 24 h and visualized by confocal microscopy.** Cells were treated with lyticase before fixation. BCO functions as the antigen; first antibody: anti-BCO produced in rabbit; second antibody: anti-rabbit produced in goat, conjugated with FITC. Bright field microscopy **(A)**, bright field microscopy merged with FITC filter **(B)**, DAPI filter **(C)**. Bar corresponds to 10 μm.

Immunofluorescence is a technique that allows the visualization of antigen-antibody interactions in cell suspensions. To this end, the BCO was used as the antigen since a first anti-BCO antibody produced in rabbit is then added, followed by a second anti-rabbit antibody produced in goat, conjugated with FITC. In this particular case, *C. albicans* was incubated with a lethal concentration of the BCO (2.38 μM) in PDB pH 7.5 medium for 24 h and observed by confocal microscopy. Calcofluor white was also added to assess the efficiency of the cell wall digestion. The results are presented in **Figure 7** and show a clear green fluorescence inside the cell. Control without the BCO was also tested and no green fluorescence was observed (data not shown). This result suggests that after 24 h of incubation, the BCO was able to cross the cell envelope and is clearly inside the cells. The calcofluor white staining indicates that not all the cell wall was efficiently digested (**Figure 7C**). However, it was sufficient to allow the cell membrane permeabilization and the subsequent entrance of the antibodies.

**FIGURE 8:**
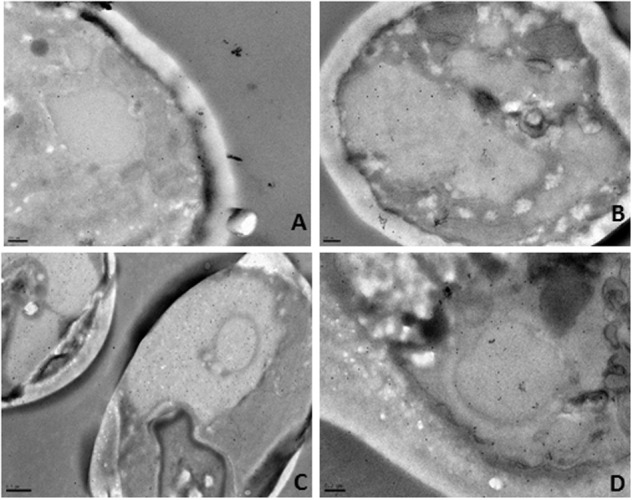
**Immunogold localization in *C. albicans* after incubation with the BCO for (A)** 2 h, **(B)** 6 h, **(C)**12 h and **(D)** 24 h, as observed by TEM. The black dots represent the localization of the BCO in the cells.

To confirm these results we next proceeded with immunogold labeling. As before, cells were exposed to a lethal concentration of the BCO for 24 h and samples were collected after 2, 6, 12, and 24 h of incubation. Each sample was analyzed by immunogold using anti-BCO produced in rat as the first antibody and a second anti-rat antibody coupled to gold particles. The immune-complexes formed were visible as little black dots when observed by TEM (**Figure 8**). At each sampling point, including time zero, the controls without the BCO were also tested and no black dots were found in none of the controls tested (data not shown). The analysis of **Figure 8** shows that the BCO progressively moves from the cell wall into the interior of the cell. Shorter incubation periods show the protein preferentially agglomerating near the cell wall (**Figures 8A,B**), while longer incubation periods show a migration of the BCO to the cytosol and even to the interior of vacuoles (**Figures 8C,D**).

### *C. albicans* Suffers Several Morphological Changes in the Presence of the BCO

**FIGURE 9:**
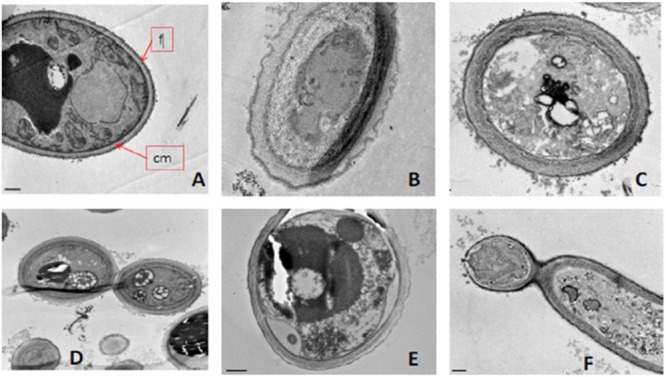
**Morphological changes after exposing *C. albicans* to a lethal concentration of the BCO as observed by TEM.** Samples were collected and visualized at times **(A)** 0 h, **(B)** 24 h and **(C–F)** 48 h. f, fibrillary layer; cm, plasma membrane.

The morphological changes undergone by *C. albicans* after exposure to the BCO, were evaluated by TEM. Cells were exposed to a lethal concentration of the BCO for 48 h and samples were collected at 0, 24, and 48 h. The results are presented in **Figure 9**. **Figure 9A** shows a section of a well preserved *C. albicans* cell, presenting a homogeneous cytoplasm, with an external fibrillar layer (f), a compact cell wall and a normal plasma membrane (cm). After 24 h incubation with the lethal concentration of the BCO, several morphological changes are visible (**Figure 9B**), mainly focused between the cell wall and the cell membrane. These changes became more clear after 48 h of incubation with a lethal concentration of the BCO, where it is visible an increased thickness at the cell wall level (**Figure 9C**), appearance of small vesicles in the periplasmatic region (**Figure 9D**), abnormal density and shape of the cell wall (**Figure 9D**), an accumulation of high density vacuoles in the cytoplasm (**Figure 9E**) and cell wall disruption, only visible in some buds (**Figure 9F**). Although the population of cells showed some heterogeneity, in general, the cells that presented more structural changes also displayed an increased size. The controls performed in the absence of the BCO showed none of these characteristics at the corresponding harvesting times (data not shown).

### Absence of Haemolytic Activity

The interaction of the BCO with mammalian red blood cells was studied by haemolysis experiments. Erythrocytes were incubated with different concentrations of the BCO, ranging from 0.04 to 4.76 μM for 1 h at 37°C. BCO showed no haemolytic effects up to 4.76 μM (data not shown), which is indicative of no detectable interference of the red blood cells.

## Discussion

The data presented above clearly show that the BCO has antifungal activity against a wide range of human yeasts pathogens, namely, *C. albicans, C. dubliniensis, C. glabrata, C. lusitaneae, C. parapsilosis, C. tropicalis and C. neoformans*. The antifungal activity of the BCO was compared to that of AMB, a commonly used human antifungal agent, and the results demonstrate that the concentrations needed to induce both cellular inhibition and cellular death are very similar to those referenced for AMB (Cantón et al., 2004; Sabatelli et al., 2006; Cordeiro et al., 2013). The BCO has already a sustained efficacy proved against phytopathogenic fungi, both under *in vitro* assays and in field trials (Monteiro et al., 2015), but this is the first report that demonstrates its activity against unicellular human pathogenic fungi.

Similarly to other antifungal compounds such as Congo red, caffeine or caspofungin, several antifungal peptides (e.g., defensins) and proteins from *Aspergillus* species are known to interfere with cell wall biosynthesis, thus weakening it by activation of the cell wall integrity pathway (CWIP) and by inhibiting chitin synthesis. Given the proven affinity of the BCO to chitin (Monteiro et al., 2015), the presence of the osmotic stabilizer sorbitol should have resulted in a significant reduction of the BCO antifungal activity if, in the end, the CWIP was its first target. However, the presence of 1.2 M sorbitol in the culture medium did not reduce the antifungal effect of the BCO suggesting that ultimately, the toxicity of the BCO is not directly related to dramatic changes caused in cell wall integrity.

All bioactivities described for the BCO up until now, were only focused on the fungal cell wall (Monteiro et al., 2015). However, chitinase and β-1,3-glucanase activities were not detected in the BCO, which apparently limits its ability to cleavage the cell wall of fungi. β-1,3-glucanase has been reported to partially digest the cell walls of *Verticillium albo-atrum*, and this degradation seems to be synergistically stimulated by chitinase (Skujins et al., 1965). Chitinases have long been reported to hydrolyse chitin from the cell walls of some fungal pathogens and non-pathogens *in vitro* (Skujins et al., 1965; Boller et al., 1983; Schlumbaum et al., 1986), and β-*N*-acetyl-D-glucosaminidase is only able to complete the degradation of chitin by hydrolyzing the soluble oligosaccharides to monosaccharides. β-*N*-Acetyl-D-glucosaminidase, one of the enzymatic activities displayed by the BCO (Monteiro et al., 2015) catalyzes the progressive release of *N*-acetyl-D-glucosamine from the non-reducing end of chitin, and together with chitinases are considered a very effective chitinolytic system on fungi (Silva et al., 2004). For example, in *Trichoderma harzianum* this system is suggested to play an important role in its potential use as a biocontrol agent against several phytopathogenic fungi (Haran et al., 1996). Chitosanase activity of the BCO is the endohydrolysis of β-1,4 linkages between *N*-acetyl-D-glucosamine and D-glucosamine residues in partially deacetylated fungal cell wall chitosan polymer. This should not to be confused with chitinase activity, which is the random endohydrolysis of *N*-acetyl-β-D-glucosaminide β(1,4) linkages in chitin and chitodextrins. Chitosan is the deacetylated version of chitin, and may possess different degrees of deacetylation (Tsigos et al., 2000). Chitosan is present in the cell wall of a small number of fungi (mainly zygomycetes), conferring structural integrity (Baker et al., 2007). *C. albicans* cell wall, like many other fungi, does not contain chitosan, so we were not expecting to see any particular rupture in the microscopic observations with calcofluor white (a specific cell wall marker for fungi, due to its chitin affinity). However, some destabilization of the cell wall structure may occur, without any visual effect by fluorescent labeling, due to its β-*N*-acetyl-D-glucosaminidase activity, at the end of the chitin polymer. In fact, the absence of visual damages at the cell wall does not imply a fully operational and structured wall, as the same can be observed even with cell wall-targeting drugs (Piotrowski et al., 2015). This might explain why the cell wall remained visually intact with calcofluor white staining, while the cells were becoming metabolically inactive, or even dead. A certain degree of disturbance in the cell wall structure seems to be required to allow the entrance of the 210 kDa oligomer. Furthermore, a clear disruption of the cell wall was observed in some bud cells. Nevertheless, the antifungal activity of the BCO does not seem to be related to the cell wall integrity, as demonstrated previously with the sorbitol test result.

Damages at the cell membrane level, measured by propidium iodide uptake, are usually indicators of cell death (Simonin et al., 2007; Choi et al., 2012), but it is also possible that some damaged cells retain the ability to recover after a short incubation (Davey and Hexley, 2011). Propidium iodide may also be used as an indicator of cell leakage, or cell permeability (Piotrowski et al., 2015). In any case, it was here demonstrated that BCO induces cell membrane damage in *C. albicans*, and this surely contributes to its inhibitory and/or lethal effect on cells. Nevertheless, and once again, this does not seem to be its primary mode of action.

Regarding the localization of the BCO in the cell, the results obtained by both immunofluorescence and immunogold labeling definitely indicate that the BCO enters the cell, although the mechanism underlying such entry is still unclear, especially considering its large molecular size (210 kDa). However, *C. albicans* is known to internalize large molecules similarly to other fungi (Riezman, 1985; Basrai et al., 1990; Helmerhorshmt et al., 2001; Oberparleiter et al., 2003; Theis et al., 2005), and such mechanism can explain the transport of the BCO into the cytosol. There are also reports of antifungal proteins being internalized by fungal cells, like NaD1 (Van Der Weerden et al., 2008, 2010), MtDef4 (Sagaram et al., 2013) and Psd1 (Lobo et al., 2007). There are several mechanisms by which these proteins can be internalized by the cell, such as receptor-mediated internalization, membrane translocation and membrane permeabilization (Vriens et al., 2014).

Many of the morphological changes observed in *C. albicans* cells upon exposure to a lethal concentration of the BCO, namely thickening of the cell wall and an increased cell size, which are the most striking features, were already observed in yeast species as a response to various stressful conditions such as halophilic stress (Kuncic et al., 2010; Gao et al., 2014) and exposure to some antifungal drugs (Ishida et al., 2009; Rueda et al., 2014). For example, in *S. cerevisiae*, the presence of salt stress causes an abnormal cell wall structure, becoming uneven and thicker, with certain areas clearly damaged (Gao et al., 2014), similarly to what was observed for *C. albicans* and the BCO. It is likely that the ultrastructural alterations described in this study also result from some chemical stress, at the osmotic level. Regarding the BCO, some experiments demonstrate that this oligomer displays a divalent-chelating activity for several cations. This biochemical property may selectively disturb the essential divalent cation metabolism of the microorganism by interfering with metal acquisition and bioavailability for crucial reactions. This chelation activity could ultimately disturb the microbial cell homeostasis and culminate in the blockage of microbial nutrition, growth and development (Santos et al., 2012).

## Conclusion

We demonstrate that after being exposed for more than 16 h to a lethal concentration of the BCO, *C. albicans* became metabolically inactive, non-viable and non-culturable. Moreover, some cells showed loss of cell membrane integrity, but with no visible rupture of the cell wall, except in bud cells. The ultrastructural alterations observed suggest that BCO imposes stressful conditions upon the fungal cell, which ultimately lead to the cell death. The disturbances observed at the cell wall and membrane seem to be just a part of a more complex mode of action of the BCO. In the future more studies are required in order to fully understand this complex mode of action, specifically its primary targets within the cell and the physiological mechanisms underlying cell death. The exceptional antifungal activity of the BCO, combined with its natural and edible origin, and the absence of haemolytic activity, provide a solid background to further investigate its potential as a novel antifungal therapeutic drug. Its current success as a phytopharmaceutical drug is already a good indicator to pursuit this goal. Furthermore, its predictable multisite mode of action suggests a low risk of inducing resistance mechanisms, which are now a major problem with other currently available antifungal drugs.

## Author Contributions

AP: Acquisition, analysis and interpretation of data for the work; Drafting of the work; Final approval of the version to be published. SM and AC: Design of the work; analysis and interpretation of data; Final approval of the version to be published. FR and RF: Important contributions on the acquisition, analysis and interpretation of data for the work. RBF: Final approval of the version to be published.

## Conflict of Interest Statement

The authors declare that the research was conducted in the absence of any commercial or financial relationships that could be construed as a potential conflict of interest.
